# Classification of advanced stages of Parkinson’s disease: translation into stratified treatments

**DOI:** 10.1007/s00702-017-1707-x

**Published:** 2017-03-24

**Authors:** Rejko Krüger, Jochen Klucken, Daniel Weiss, Lars Tönges, Pierre Kolber, Stefan Unterecker, Michael Lorrain, Horst Baas, Thomas Müller, Peter Riederer

**Affiliations:** 1 Luxembourg Centre for Systems Biomedicine (LCSB), University of Luxembourg, Esch-Sur-Alzette, Luxembourg; 2 Centre Hospitalier de Luxembourg (CHL), Luxembourg, Luxembourg; 3 Molecular Neurology, University of Erlangen, Erlangen, Germany; 4 Department for Neurodegenerative Diseases and Hertie-Institute for Clinical Brain Research, Center for Neurology, University of Tübingen, Tübingen, Germany; 5 Department of Neurology of the Ruhr-University Bochum at St Josef-Hospital, Gudrunstrasse 56, 44791 Bochum, Germany; 6 Center of Mental Health, Clinic and Policlinic of Psychiatry, Psychosomatics and Psychotherapy, University Hospital, Würzburg, Germany; 7 Nervenarztpraxis Gerresheim, Düsseldorf, Germany; 8 Department of Neurology, Klinikum Hanau GmbH, Hanau, Germany; 9 Department of Neurology, St. Joseph Hospital Berlin-Weissensee, Berlin, Germany

**Keywords:** Advanced Parkinson's disease, Stratification, Personalized medicine, Invasive therapies

## Abstract

Advanced stages of Parkinson’s disease (advPD) still impose a challenge in terms of classification and related stage-adapted treatment recommendations. Previous concepts that define advPD by certain milestones of motor disability apparently fall short in addressing the increasingly recognized complexity of motor and non-motor symptoms and do not allow to account for the clinical heterogeneity that require more personalized approaches. Therefore, deep phenotyping approaches are required to characterize the broad-scaled, continuous and multidimensional spectrum of disease-related motor and non-motor symptoms and their progression under real-life conditions. This will also facilitate the reasoning for clinical care and therapeutic decisions, as neurologists currently have to refer to clinical trials that provide guidance on a group level; however, this does not always account for the individual needs of patients. Here, we provide an overview on different classifications for advPD that translate into critical phenotypic patterns requiring the differential therapeutic adjustments. New concepts refer to precision medicine approaches also in PD and first studies on genetic stratification for therapeutic outcomes provide a potential for more objective treatment recommendations. We define novel treatment targets that align with this concept and make use of emerging device-based assessments of real-life information on PD symptoms. As these approaches require empowerment of patients and integration into treatment decisions, we present communication strategies and decision support based on new technologies to adjust treatment of advPD according to patient demands and safety.

## What is advanced Parkinson’s disease?

The traditional classification and disease progression of Parkinson’s disease (PD) orient on disease milestones that can be most obviously followed along motor domains. In this sense, the topography and severity of segmental motor symptoms, followed by more bilateral segmental involvement, finally appearance of gait disturbance, postural impairment and bedridden immobile states provide well defined but also in some way broadly scaled categories of disease stages. Although this and similar classifications are valuable to approximate and describe the motor severity over time, the classifications fall short to comprehensively describe and characterize the full, continuous and multidimensional spectrum of disease-related motor and non-motor symptoms. In recent years, diverse non-motor domains, quality of life, psychosocial burden and stigma have received major attention as determinants of PD disease course and outcome parameters of clinical trials (Deuschl et al. 2006; Schuepbach et al. 2013). Diversity in neurodegeneration patterns and involvement of several neurotransmitters and their contribution to motor and non-motor symptom parallel the phenotypic variability (Sauerbier et al. 2016a; Titova et al. 2016).

Characterizing PD patients on such broad scales is essential, since the phenotype of individual patients varies substantially. This diversity leads to ultimate differences in patients’ therapeutic requirements, and will very differentially affect patients’ subjective well-being, self-perceived disease-related impairments, and health-related quality of life.

Thus, the following questions remain: when one would talk from advanced PD (advPD)? Would it be the presence of a particularly severe symptom? Would it be the combination of different symptoms as red flags? Would it be more rapid progression? What is the respective threshold for considering treatment escalation? And, who would finally decide that an advPD stage was reached? A general practitioner, an expert neurologist? Or should the patient’s self-perception even prompt severity categorization?

Indeed, there is no uniquely accepted operationalization to this end and, thus, the additional efforts are justified to address this yet unmet need (Antonini et al. 2015; Luquin et al. 2017). An approximation towards a unique definition of advPD was strongly proclaimed recently—in particular to ensure referral of the right patient to an adequate therapeutic regimen at the right time. This should help to refer patients to specialized centers for introduction of advanced therapies like neurostimulation or continuous dopaminergic pump therapies, following recommendations from randomized controlled trials (Odin et al. 2015; Olanow et al. 2014; Deuschl et al. 2006). Owing to this perspective, classifying a PD patient as “advanced” would sensitize patients, caregivers, and non-specialized general practitioners, as well as specialized expert neurologists to prompt therapy referral at the right time—ultimately right before the patient would suffer for years from an unaddressed therapeutic gap by missing adequate and effective therapy, which would put the patient at risk for irreversible and handicapping sequelae (Odin et al. 2015). This needs to include both motor and non-motor domains and implies a more holistic view of PD (Sauerbier et al. 2016a). Further hallmarks of the late disease stage clearly outweigh a focus on motor and non-motor complication along disease progression. This mainly incorporates cognitive incompetence, uncontrolled psychiatric issues like psychosis, or resistant axial motor symptoms like imbalance or gait impairment that finally would imply loss of self-dependence and increasing dependence on care (Krüger et al. 2015; Weiss et al. 2013).

Another critical and unaddressed issue when guiding therapy along evidence-based medicine is the potential bias from classical clinical trials, since study cohorts that are generally based on strict inclusion and exclusion criteria and, therefore, cannot sufficiently represent the overall PD population as a whole. In other words, such trials generally under-represent ‘common’ PD patients with multiple co-morbidities. However, these patients constitute the most relevant and demanding treatment group in routine daily practice (Sprenger et al. 2014). Therefore, evidence-based medicine deduced from classical trials may fail to translate into daily life clinical practice in a relevant proportion of patients—notwithstanding the important merits of such high-quality trials.

Current treatment of PD is characterized by polypharmacy and, therefore, implies potential complications through interactions between different medications. Moreover, different pharmacokinetic aspects have to be considered. First of all, gastric emptying in patients with PD is slowed in advanced stages of the disease with a relevant influence on absorption (Nyholm 2006). Regarding the biotransformation, most anti-parkinson drugs are metabolized hepatically and only amantadine is mainly excreted unchanged via the kidney (Hiemke et al. 2011). Practical aspects of therapy of advPD focus on treatment optimization under changing pharmacokinetic and pharmacodynamic conditions (Müller 2012).

Regarding the risk of side effects by a drug–drug interaction, it must be considered that PD predominantly is a disease of the elderly (Wright Willis et al. 2010). Therefore, most of these patients suffer from different diseases, and in the consequence often enough are treated with a whole range of different medications (Csoti et al. 2016). Polypharmacy in the elderly is commonly performed with the number of drugs increasing in parallel with patient’s age (Cascorbi 2012). Besides bromocriptine as CYP3A4 inhibitor, anti-parkinson medication itself has no properties of pharmacokinetic induction or inhibition (Hiemke et al. 2011). Although anti-parkinsonian medication generally seems to play no relevant role in drug–drug interaction described on neurology wards (Namazi et al. 2014), drug–drug interaction studies in PD give suggestions for treatment in patients with comorbidities like arterial hypertension (Bitner et al. 2015) or diabetes mellitus and other internal medicine diseases (Csoti et al. 2016). These complex interactions in multi-morbid advPD patients justify and add value to observational studies related to novel treatment options that may contribute real-life information on the usefulness of new therapeutic options (Pålhagen et al. 2016). Here, registers and collection of real-life data are encouraged to obtain larger and unselected data pools and to complement evidence-based medicine.

Together, patient demands and neurologist reasoning on clinical care and therapeutic decision are highly complex and multidimensional. Owing to the complexity of clinical decisions, classical clinical trials that are guiding elements of evidence-based medicine can justify clinical decision making on a group level according to specifically defined criteria, but may fall short for an individual patient given the complexity and interindividual variability of phenotype and patient demands. This consideration prompted the so-called ‘precision medicine’ concept (Robinson 2012), i.e., it is expected that an in-depth precise phenotyping would finally guide the physician towards the best individual care for an individual patient and his/her personal needs and requirements. Integration of both objective and subjective surrogates—according to the so-called ‘patient-and-physician partnering perspective’ as part of the Parkinson Net in the Netherlands (Gray et al. 2016)—might finally imply a different operationalization of the term “advPD”. In this sense, advPD would not adhere too tightly on disease milestones and progression, but might rather reflect distinct phenotype scenarios across the very broad-scaled and multidimensional PD phenotype (including motor, non-motor, quality of life, psychosocial, contextual aspects). If so, advPD would rather point to critical phenotypic presentations needing therapeutic adjustment (as opposed to a pure ‘disease stage approach’). In such an ideal and highly differentiated framework, deep phenotyping would prompt and differentiate clinical decisions along the multimodal features to guide therapy towards its utmost precision and safety.

## Strategies to define diseases stages

As already stated in the introductory part, there are many possibilities to classify or subtype PD: age of onset, clinical phenotypes (motor and non-motor), disease severity or neuropathological alterations. We here present the current scientific knowledge of frequently used classifications and thereby want to provide the basis for subsequent stratification of patients, that is needed to identify the optimal treatment for the individual patient. We acknowledge that a classification or staging of PD as a heterogeneous neurodegenerative disease is to some extent artificial, but still consider it important, especially in view of the emerging highly specific causative treatment concepts.

### Disease onset of PD: juvenile, early and typical forms

A frequently applied classification of PD depends on the time point of disease onset. *Juvenile PD* develops until an age of 20 years, *early onset PD* until 40 years (some authors enlarge the time frame until age 45). Thereafter, development of disease is regarded as a normal onset. In the case of juvenile or early onset PD before the age of 35 years, a further genetic analysis, even in the absence of a positive family history, as typically related to an autosomal recessive inheritance with unaffected parents, is worthwhile (Sheerin et al. 2014). Early onset patients typically present with a more benign disease course and are less frequently subject to cognitive impairments; however, motor fluctuations are typically observed and can justify interventional treatment options within the advPD concept (Hassan et al. 2015) Generally, these forms of PD are typically rare and as a chronic-progressive neurodegenerative disease, most patients are diagnosed at a rather advanced age. Thus, at the age of 65, the incidence amounts to approximately 50 in 100,000, at age 75–150 in 100,000, and at age 85–400 in 100,000 (de Lau et al. 2006; Pringsheim et al. 2014). In an elaborate approach to approximate the prevalence of prodromal PD—which might be present already about 10 years before clinical diagnosis—Berg et al. calculated a prodromal prevalence of 0.5% at age 55, 1.5% at age 65, and 4% at age 75 (Berg et al. 2015). This means that at the age of 75 years about 1% of the population will be diagnosed with PD, but an additional 4% will already have prodromal PD and might develop the classical motor symptoms within the next 10 years. With these numbers, the strong impact of PD for our aging society becomes obvious.

### Overall clinical disease classification and motor scales: HY stage and UPDRS scale

In an early study of PD patients between 1949 and 1964, Margaret M. Hoehn and Melvin D. Yahr classified patients based on their degree of disability into five categories, the widely used HY stages I–V. Among all patients classified accordingly, the proportion of those who were severely disabled or dead within 5 years of disease onset was about 25 percent. After follow-up for 5–9 years, this percentage increased to 67, and to 80% after 10–14 years. Only a small group of patients showed a slower disease progression and maintained balance and postural stability for more than 10 years, some even lacking severe disability more than 20 years later (Hoehn and Yahr 1967). In a more recent study of 142 PD patients who had been long-term followed from 2000 to 2012, about 77% had an advanced outcome at 10 years after diagnosis which was mostly due to dementia or postural instability. Most causes of death were not directly related to PD but consisted in pneumonia, cancer, cardiac disease and other reasons (Williams-Gray et al. 2013). It is important to note that the transition from HY stage II to III marks a milestone in PD, because disease impairment with gait and balance difficulties results in overt disease disability and restricts gait-dependent activities.

While strengths of the HY scale are its wide utilization and acceptance as well as a correlation to standardized scales for motor impairment, disability, and some aspects of quality of life, it has also weaknesses. Of these, the scale’s mixing of impairment and disability and the non-linearity of the scale are most important (Goetz et al. 2004). Therefore, more differentiated scales with focus on motor impairment including the cardinal symptoms of PD (hypo-/bradykinesia, rigidity, rest tremor, postural instability) have been developed and can additionally be applied. Here, the *Unified Parkinson Disease Rating Scale (UPDRS)* or a modified form as proposed by the MDS (*MDS*-*UPDRS*) is available (Movement Disorder Society Task Force on Rating Scales for Parkinson’s Disease. 2003; Goetz et al. 2004, 2008). Basically, the scale contains four domains consisting of cognition and mood (part I: non-motor experiences of daily living), activities of daily living (part II: motor experiences of daily living), motor examination (part III), and motor complications (part IV). It has a high validity for rating in PD as was shown after an elaborate clinimetric test of the scale (Goetz et al. 2008). Based on the MDS-UPDRS scores, cutoff points to sub-classify PD patients were proposed recently. Here, cutoffs in each of the four parts of the scale were defined for mild, moderate or severe stages (cutoff points between mild/moderate and moderate/severe levels as follows: Part 1: 10/11 and 21/22; Part 2: 12/13 and 29/30; Part 3: 32/33 and 58/59; and Part 4: 4/5 and 12/13). This can help to better stratify disease severity of PD, identify clinical red flags for advPD and assign treatment strategies with respect to overall disease progression and with a focus on motor symptoms (Martinez-Martin et al. 2015).

### Non-motor symptoms and PD subtypes

Parkinson’s disease (PD) has traditionally been considered a motor system disorder, but it is now widely regarded as complex disorder with distinct clinical features that also include neuropsychiatric and non-motor manifestations (Chaudhuri and Sauerbier 2016). The most relevant non-motor features comprise cognitive dysfunction and dementia, psychosis and hallucinations, mood disorders including depression, anxiety, and apathy/abulia, sleep disturbances, fatigue, autonomic dysfunction, olfactory dysfunction, gastrointestinal dysfunction, pain and sensory disturbances as well as dermatologic findings (seborrhea). Although these symptoms are in part included in the MDS-UPDRS scale, more specific scales exist which exclusively evaluate non-motor function such as the patient self-questionnaire NMS-Quest (Chaudhuri et al. 2006) or the physician-assisted NMS Scale (Chaudhuri et al. 2007). These scales capture the non-motor burden of disease and enable a more holistic view on PD, since non-motor symptoms were shown to strongly influence overall severity of disease in PD patients (Chaudhuri et al. 2013, 2015). Moreover, recent classifications of advPD are clearly referring to non-motor symptoms, e.g., symptomatic dysautonomia (including orthostatic symptomatic hypotension), excessive daytime sleepiness, hallucinations and cognitive impairment (Luquin et al., 2017).

While an effort to classify PD according to motor symptoms into different predominant phenotypes such as tremor-dominant and non-tremor-dominant (postural instability gait disorder/akinetic-rigid) subtypes has already been undertaken, such a classification has recently moved into focus also for non-motor phenotypes. Here, the following non-motor subtypes are distinguished: cognitive, neuropsychiatric (apathy, depression/anxiety), sleep (REM sleep behavior disorder), (central) pain, fatigue, autonomic (gastrointestinal tract dysfunction, genital-urinary disorders, symptomatic hypotension), and “Park weight” (combined with olfactory dysfunction and dyskinesia) subtype (Marras and Chaudhuri 2016; Sauerbier et al. 2016b). Interestingly, the non-motor symptom patterns reflect phenotypes which can be characterized by dominant involvement of either neocortical, olfactory/limbic or brain stem areas and thus demonstrate the strong link to the underlying neuropathological and biochemical (e.g., cholinergic, serotonergic, opioidergic, adrenergic) disturbances (Marras and Chaudhuri 2016).

Of course, the motor and non-motor symptoms often overlap to some extent and clinically defined PD subtypes are unlikely to be distinct non-overlapping entities. Much more likely, they represent typical phenotypes within a multidimensional spectrum resulting from variable contributions of several simultaneous pathological processes. In this context, a frequent association of axial motor symptoms (i.e., gait disturbances and falls) with cognitive impairments has been observed that shows the implication of overlapping functional brain circuits (Amboni et al. 2013; Hausdorff et al. 2006). This co-occurrence implicates different neuronal structures in the brainstem, cerebellum and cortex and, therefore, translates into the pathophysiological concept presented in the following section.

### Neuropathological staging

With the help of a precise description of motor and non-motor phenotypes, a correlation with neuroanatomical structures and subsequent neuropathological alterations becomes feasible. In general, idiopathic PD is regarded as a slowly progressive disease spreading within the nervous system, which explains that first symptoms are often very difficult to pinpoint within an individual patient. Through very detailed neuropathological analyses of post-mortem material of PD patients, Braak et al. (2003) and Beach et al. (2009) described distinct pathways of neuronal degeneration, Lewy body pathology and spreading of disease in the CNS (Braak et al. 2003; Beach et al. 2009). Braak suggested that the disease process including synucleinopathy with Lewy body deposition may start in non-dopaminergic structures in the periphery and then spread in an ascending way to the olfactory bulb and lower brainstem which could explain early autonomic disturbances and hyposmia (Braak stages I/II). Then, brainstem synucleinopathy was found to migrate rostrally to the substantia nigra pars compacta and other neuronal clusters of the midbrain and basal forebrain and classic motor symptoms appear (Stages III/IV). Ultimately, the telencephalic cortex of the temporal and frontal lobes was shown to be involved (Stages V/VI) (Braak et al. 2003). According to this concept, advPD correlates with the implication of neocortical structures implying cognitive impairment. Interestingly and in accordance with the concept of a pathophysiological process affecting dopaminergic and non-dopaminergic structures, patients with a faster disease progression towards advPD present with earlier cognitive impairment and postural instability (Van Der Heeden et al. 2016).

Recently, the validity and predictive utility of Braak staging have been questioned because the extent of synucleinopathy does not correlate with clinical disease severity and may also be present in healthy individuals (Parkkinen et al. 2005). Furthermore, the very common asymmetry of clinical symptoms is not reflected in disease pathology (Riederer and Sian-Hülsmann 2012), not to mention predominantly cognitive disease courses such as Lewy body dementia, which very early on manifests with cortical involvement (Halliday et al. 2011; Jellinger 2012). Thus, novel aetiopathogenic hypotheses of PD emerged, among them the so-called “threshold theory”. It suggests that the functional threshold is lower for the emergence of early peripheral and autonomous symptoms before the appearance of the classical motor symptoms of PD because the functional reserve of the midbrain dopamine and integrated basal ganglia motor systems to control movement is much larger than, e.g., for the enteric nervous system (Engelender and Isacson 2017). Through further ongoing analyses, it will be shown which concept is more robust or if these two should be harmonized to some extent.

### Challenges to classify disease stages at the boundary of advPD and atypical parkinsonism

During disease progression and based on the predominant motor and non-motor features associated with advPD, the separation from atypical parkinsonism (AP) may be difficult and overlap syndromes like ‘minimal change’ multiple system atrophy (MSA) or progressive supranuclear palsy with predominant parkinsonism (PSP-P) have been described (Petrovic et al. 2012; Respondek and Höglinger 2016). AP includes a heterogeneous bunch of syndromes, all characterized by clinically manifest parkinsonism in combination with other clinical features and a poor therapeutic response to dopaminergic medication. Only post-mortem analyses can clearly differentiate from advPD, as their neuropathology is characteristically different: in MSA, alpha-synuclein accumulation is found and defines an alpha-syncleinopathy as PD, but mainly in glial cells as cytoplasmic inclusions (coiled bodies). In contrast, PSP and corticobasal degeneration (CBD) are referred to as tauopathies due to characteristic intraneuronal tau aggregation and some TDP-43 proteinopathies might also develop clinical parkinsonism (Dickson 2012; Siuda et al. 2014; Stamelou et al. 2013).

In all parkinsonian syndromes, correct diagnostic classification is essential for the definition of treatment options and the accuracy of any prognosis. However, even in experienced centers, the diagnosis of PD and its diagnostic differentiation from AP have poor reliability and are often incorrect, if exclusively based on clinical criteria. In a number of clinical studies, there is an error rate of at least 10–30% in such cases. Diagnostic accuracy can improve by consequent use of standardized diagnostic instruments such as the Queens-Square-Brain-Bank (QSBB)-criteria, including its supportive signs. QSBB-criteria include mainly motor symptoms and, therefore, non-motor symptoms are under-represented in these criteria. However, there is still a remarkable difference in the diagnostic accuracy between experts and non-experts, even if such standardized criteria are used, and also among experts a notable percentage of misdiagnosis has been observed in longitudinal observations (Hughes et al. 1992; Postuma et al. 2015; Rizzo et al. 2016).

Thus, differentiation of advPD from AP still remains a diagnostic challenge, especially for slowly progressive forms of AP that may present substantial overlap with advanced stages of PD, e.g., in terms of falls, dysphagia and cognitive impairment (Luquin et al. 2017). In these rare forms, even dyskinesia can be observed in patients with MSA that goes beyond phasic dystonia. These can present as choreatiform and generalized dopamine-induced dyskinesia and, therefore, complicate the proper diagnosis of AP (Petrovic et al. 2012). The differentiation between advPD and AP is critical, as advPD typically defines the threshold to implement intensified, typically interventional therapies like pump-systems or DBS. However, patients with AP have no sustained response to dopaminergic or neuromodulation treatments and, therefore, the peri-interventional risk is not justified. This was recently underscored in series of neuropathologically confirmed cases with benign, slowly progressive MSA, who underwent STN-DBS. Only a subset of these patients showed a short-term benefit from DBS that was rapidly counteracted by severely disabling symptoms related to MSA (Meissner et al. 2016).

In this context, technical tests might further improve the quality of differential diagnosis. Autonomous tests, such as tests for cardiovascular, urinary, thermoregulatory or gastrointestinal dysfunction can be helpful for the diagnostic differentiation PD versus AP. Due to a marked overlap, the combination of several tests such as urodynamic investigation, tests for orthostatic dysregulation, RR-intervals and sympathetic skin response can contribute to support the correct diagnosis.

In MSA, olfactory dysfunction is also found in a number of studies, but has led to controversial results with respect to its frequency and severity. Yet, in terms of specificity, in PSP and CBD as well as in vascular parkinsonism the olfactory function seems to be far less compromised and may serve for differential diagnosis towards PD but the result of smell tests can be biased by the fact that slight to moderate olfactory loss is also found in 20–50% of elderly healthy subjects (Haehner et al. 2014; Takeda et al. 2014).

Finally, imaging is probably the most frequently used ancillary examination to differentiate PD from AP. Besides structural imaging to visualize typical signs for MSA-p (e.g., pontine and putaminal atrophy, hyperintense putaminal rim, hyperintense middle cerebellar peduncle or the hot-cross bun sign), PSP (midbrain atrophy and an enlarged third ventricle) and CBD (asymmetric cortical atrophy), functional brain imaging has been applied. Single photon emission tomography (SPECT) with various ligands can help to distinguish PD from AP, e.g., IBZM-SPECT is still sometimes used for the differentiation PD vs. MSA-p but has not fulfilled preliminary expectations since clinical practice has shown that the results are not sufficiently reliable. However, cardiac MIGB-SPECT has been proven as a more reliable tool for the identification of AP in early stages of parkinsonism (Chun et al. 2009).

In summary, there is yet no test available, which has sufficient sensitivity/specificity for the accurate clinical diagnostic separation of advPD vs. AP, when it is used exclusively as an isolated procedure. However, the diagnostic accuracy can be improved by the combination of the above-mentioned ancillary methods in addition to the physical examination. The exact diagnostic classification is important for the individual prognosis and patient’s counseling towards interventional therapies, even if the diagnosis of AP has limited therapeutic consequences, since treatment is restricted to symptomatic procedures which are identical in different forms of AP conditions (Garcia-Ruiz et al. 2014; Reichmann et al. 2016).

Future deep phenotyping approaches in longitudinal cohorts may help to further differentiate between advPD and AP and define mechanism-based therapeutic approaches that can be applied to different clinical entities, e.g., PD and MSA as synucleinopathies as currently investigated for green tea component Epigallocatechin-gallate (EGCG) that interferes with alpha-synuclein aggregation in vitro and in vivo (Levin et al. 2016).

## New treatment targets in advanced Parkinson’s disease

Motor and non-motor symptoms acquire distinct characteristics in advanced stages of PD that differ from early stages (Olanow et al. 2009). It is important to note that not only the symptom patterns become more complex with progressing disease stages, but also more individualized. Additionally, therapeutic options become more complex for advPD, as there are surgical therapies, pumps, patches or individualized combinations of different treatments options. Thus, patient populations become more complex, both for standardized assessments and clinical care. Targeted therapies require substantial preselection of patients based on their symptom patterns and outcome parameter requires highly specialized questionnaires and examination strategies. Pathomechanistic independency or confounding between these symptoms, as well as their comparative responsiveness to, e.g., dopaminergic treatment is only partially understood. Also, preselection based on only a limited number of symptoms can lead to highly conserved patient cohorts within clinical trials that do not allow the translation into other patient group with different symptom patterns. Thus, an easy transfer from clinical studies with the highest evidence level to patients at the same (advanced) disease stage within standard clinical management becomes increasingly limited. Even though focused and standardized clinical diagnostic queries and examination protocols are able to assess the individual symptoms, their contribution to the activities of daily living and patient centered quality of life related outcomes is only partially understood. Huge patient cohorts and objective targets are required to understanding this complexity between individual symptom patterns, highly focused assessment strategies, pathomechanistic causal relationships, and resulting consequences for the overall quality of life in patient-centered clinical management and care concepts.

Within the emerging area of healthcare technology, developments in PD objective assessment strategies become increasingly developed and studied focusing on the variety of motor and non-motor symptoms in PD (Klucken et al. 2013; Maetzler et al. 2016). In contrast to novel imaging strategies that become more sensitive to structural and functional neurodegenerative changes, wearable technologies become increasingly inexpensive and allow for the objective assessment of distinct symptoms in PD (Espay et al. 2016). Even though most of the new technologies still lack the required technological readiness level (Sánchez-Ferro et al. 2016), they clearly pave the way for a substantial change in diagnostic and treatment paradigms. Two different concepts have to be distinguished on how technology supports both clinical care and studies: while several systems aim to improve the accuracy and comparability of standardized clinical assessment tests already used especially in clinical studies (e.g., improving tremor rating of related items of the UPDRS), others aim to assess new clinically relevant targets from the everyday life of the patient reaching out to individualized continuous monitoring concepts. Ideally, a new technology would present a sensor—or a group of sensors—that assesses all the relevant symptoms of an individual patient continuously resulting in an individualized pattern and objective score exactly predicting the health-related quality of life. It is evident that this predicted scenario is likely to be substantially more complex than the above-mentioned stratification options in advanced PD. Nevertheless, these increasingly easy-to-use assessment strategies allow handling this complexity using modern big data mining strategies and machine learning support.

A good example is the concept supported by mPower: a relative simple smartphone-app assesses with short questions or easy motor tasks a complex pattern of features for each patient. Since it is easy to download and install, already over 9.500 patients have registered and include their data (Bot et al. 2016). The implementation of novel technologies ultimately has the potential to provide patterns of symptoms extracted from real-life patient scenarios and allows for a more direct and active participation of patients to research programmes, which might improve their quality of life (Van Uem et al. 2016). Today a great number of different technologies for domestic monitoring of motor symptoms do exist, ranging from wearable sensors to non-wearable devices or gait labs (Godinho et al. 2016). Non-motor symptoms like sleep quality, skin humidity or cardiovascular function can also be monitored, but still need development to improve practicability and consequently adherence of the patient to the device-based assessment (Espay et al. 2016).

Nevertheless, a substantial amount of validation work is required, in particular, because the patient inclusion criteria are not limited or supervised by trained movement disorder specialists. Also, it is not clear, which of these “new” target parameters is able to measure therapeutic effects in each dimension. In addition, regulatory aspects for medical technologies as well as data safety and privacy concerns have to be developed and met. This also requires new IT-based communication strategies that connect and harmonize the team of multidisciplinary care, and modular selection strategies for distinct technologies assessing the symptom pattern of each individual patient. If these goals are reached, it is possible to provide the best care concept for patients within standardized clinical management and at the same time provide stratified real-life targets for clinical studies. The coming years have to prove the clinical validity and applicability within this emerging field of technology in PD (Pasluosta et al. 2015).

## Stratification for therapeutic outcomes

Based on the increasingly recognized heterogeneity of PD—not only in terms of underlying genetic and/or environmental causes, but also in terms of clinical presentations—there is an emerging need for better definitions of subtypes of PD that allow to assign treatments and shape therapeutic approaches according to the best response. As there is still no established neuroprotective treatment option that is able to intervene with the chronic neurodegenerative process, most benefit for the patients in terms of quality of life can be currently achieved by providing access to best symptomatic treatment. This is also reflected by the fact that clinical trials focus on more meaningful parameters in terms of primary and secondary outcomes (Mestre et al. 2015; Schuepbach et al. 2013). Complications of symptomatic pharmacological treatment of PD like dyskinesia remain a significant problem and several recent trials failed to efficiently target dyskinesia at phase III level (Mestre et al. 2014; Orloff et al. 2009). Therefore, the translation of novel drugs into successful trials requires the definition of ‘clinically important change’ that goes beyond the application of clinical rating scales and aligns with the patient’s observation, e.g., of remission and perception of dyskinesia.

Similarly, quality of life is a relevant clinical outcome parameter and in studies investigating the role of deep brain stimulation (DBS) in advPD (Deuschl et al. 2006). The results of the EARLYSTIM study supported this concept and indicated that DBS was superior to best pharmacological treatment in younger PD patients with early motor fluctuations (Schuepbach et al. 2013). Therefore, age or disease stage of patients may represent first clinical stratifiers for more adapted symptomatic treatment approaches. However, these criteria only apply at the group level and more advanced strategies to predict therapeutic outcomes that include additional, objective traits for personalized treatment recommendations are highly warranted.

Here, genetic stratification has already proven effective in the treatment of different forms of cancer, either by defining tumor subtypes more or less responsive to therapies (e.g., in ovarian cancer by genotyping BRCA1/2 variants) or by defining a profile of the individual metabolizing capacities in terms of pharmacogenomics (Relling and Evans 2015). Recently, first pilot studies were published for PD patients and correlated positive treatment outcomes for symptomatic pharmacological or interventional therapies with specific genotypes in candidate genes.

In a first study, DNA samples from 692 participants of the ADAGIO study that represented the largest clinical trial of early stage PD patients under monotherapy with rasagiline were genetically stratified for 197 genetic polymorphisms from 20 candidate genes (Masellis et al. 2016). The candidate genes were chosen by their involvement in rasagiline’s mode of action or metabolism or based on previously reported genetic association with PD in genome-wide association studies (GWAS). The authors found a polymorphism in the dopamine D2 receptor gene as predictive for a meaningful clinical response to rasagiline treatment (Masellis et al. 2016). This effect was not associated with the rate of symptom progression during the trial period. As the beneficial genotype of the DRD2 receptor gene was associated with structural changes leading to a ‘short isoform’ of the DRD2 receptor, the authors speculated that increased dopamine levels due to monoamine oxidase B inhibition by rasagiline would lead to a greater increase in cortico–striato–thalamo–cortical motor activity resulting in improvement in PD symptoms.

Another study investigated the contribution of genes to the positive therapeutic outcome in PD patients treated with DBS. Therefore, polymorphisms in the alpha-synuclein gene and LRRK2 gene were investigated in a cohort of 85 PD patients treated with DBS in the subthalamic nucleus (STN) and followed for more than 2 years after implantation. The candidate genes were chosen based on the fact that both consistently found associated with PD in large GWAS studies from different populations worldwide (Simon-Sanchez et al. 2009; Nalls et al. 2014). Interestingly, a certain allele of the alpha-synuclein polymorphism predicted a positive outcome of DBS in a dose-dependent manner with homozygous carriers showing the most pronounced benefit (Weiss et al. 2016). The same genetic variant was linked to reduced expression of the PD-associated alpha-synuclein protein in different brain areas assessed by different post-mortem studies (Linnertz et al. 2009; Fuchs et al. 2008). This may indicate that the burden of alpha-synuclein accumulation could relate to the integrity of the basal ganglia loops that are critical for effective neuromodulation. In support of this hypothesis, the same alpha-synuclein polymorphism that was associated with reduced expression of alpha-synuclein in human brains was associated with PD without cognitive impairment in a recent association study on PD patients with and without dementia (Guella et al. 2016). This further supports the concept of genetic variants in the alpha-synuclein gene as potential tools for stratification in clinical trials.

The previous findings on pharmacological or neuromodulation therapy still require validation in independent cohorts, which are difficult to find due to the clinically well-characterized cohorts that served as starting points for the pilot studies and, therefore, should stimulate efforts for deep phenotyped patient cohorts for translational research. These cohorts will be also critical for the development of clinical trials that focus more on individual not average therapeutic response.

Current medications provide efficacy only in a subset of patients, e.g., only 1 in 50 patients benefit from statins used to lower cholesterol (Mukherjee and Topol 2002). The underlying clinical trials collected a handful of parameters from a large number of study participants. Future trials for more precise medical treatment approaches will be designed to capture a large number of different parameters, but only in limited number of participants to allow for assessing the individual patient’s response to therapy. The growing interest in ‘omics’ assays that define the individual characteristics on the molecular level and also include genetic profiles and metabolomics will help to avoid side effects and predict more precisely drug–drug interactions. The latter are frequently observed in PD as advanced stages in patients with typically advanced age currently imply polypharmaceutical approaches.

### Practical aspects for the implementation of advanced treatments

In the past few years, the treatment of PD has become increasingly complex and it is expected to be more individualized in the future, which implies novel strategies for best practices to define and convey best treatment options to patients with advPD. Current guidelines are a helpful tool in the diagnostics and therapeutic decision making in the early stages of disease; however, there is not enough reliable information on how to implicate the suggested strategies in the everyday neurological practice. In addition, there is little specific information on possibilities of influencing the course of disease progress. In addition to the usage of the oral medication in the early stages of the disease, there has been an increase in application of the interventional therapies such as deep brain stimulation and pump therapies. These highly specific treatment options are mostly implemented in specialized clinics or practices for movement disorders. Here, the optimal timing for initiating advanced therapies to improve the quality of life and prevent complications is critical and requires an early information of patients and caregivers about the later stages of the disease with its complications.

To avoid and/or to reduce anxiety and rejection, sufficient and regular explanation about the possible therapies at early stages of disease can widen opportunities for overall therapeutic strategies. The practicing neurologists should be involved in rounds for movement disorders as part of the extended therapeutic concept (Krüger et al. 2015). Pharmacists and medical associations also serve as an important source of information for patients, less so the peer groups and the health insurances. It is not clear to what extent this applies to PD patients. Since the therapeutic decisions for PD patients are seldom based on scientific studies, it is important to provide information to all the involved persons.

To adapt information on therapies to the specific requirements of the patient is important and will reassure engagement of the patient. The passing on of information is critical and has to be addressed appropriately, in order to achieve adequate adherence to therapy and to deal efficiently with possible complications. Here, different types of patients may require specific approaches. The young informed patient is often shocked at delivery of diagnosis and, therefore, needs extended information. Management of these patients is usually not problematic; however, the digital information overflow can pose obstacles and lead to anxieties. Therefore, it is essential to offer low-threshold and frequent explanatory briefings. This is the basic principle in the therapy of PD: the extensive information about the disease itself (motor/non-motor symptoms), the course of disease and its therapy has to be often repeated and explained. As in many chronic diseases, suppression tends to be a common psychological strategy in coming to terms with the disease. Partner of the patient is often the one to communicate with the physician. It is of great importance to involve the patient in the conversation. In later stages of disease, symptoms can be misinterpreted and increase of side effects and complications can occur due to self-regulation of the dosage of medication. The patient management in such cases can be time consuming. Here, specialized Parkinson nurses are available to answer disease-related or care-related questions and to manage administrative issues.

Recent technological developments allow for the integration of interactive information platforms in patient information and feedback. Appropriate feedback mechanisms and evaluation system in corresponding online portals have to be available. Implementing interactive platforms in the practice setting would provide more transparency and simplify feedback and evaluation for more efficient patient empowerment and allow for the implementation of interactive communication in the ambulatory patient care in the near future (Chiauzzi et al. 2016).

## Outlook

As PD is increasingly recognized as a heterogeneous disorder, and especially the advanced stages of the disease with complex interplay of motor and non-motor symptoms demand for more individual adaptation, careful drug titration and combination of therapies. As advPD patients are typically older and subject to multiple co-morbidities, classical drug targeting strategies derived from large clinical trials in unselected patients do not translate directly into clinical practice. It is, therefore, not surprising that within this framework the concept of disease modification has more or less failed from the clinicians’ point of view. Therefore, novel approaches that take into account the heterogeneity of advPD and translate into novel clinical study concepts are required.

To date, etiology of sporadic PD is still unknown. As an example, it is far from clear whether increased nigral and striatal Lewy Body (LB) occurrence is a specific process responsible for onset of PD or whether it is the result of a secondary pathological process. Actually, the term PD describes a concept for an entity of different subtypes. There is a certain overlap between each of them and not all share the same neuropathologically driven concept of increased LB presence as essential feature of PD (Braak et al. 2003; Beach et al. 2009). In this regard, current research on genetics helped to define rare forms of monogenic PD and rare genetic variants with significant effects like mutations in the glucocerebrosidase (GBA) gene; however, genome-wide association studies (GWAS) do not yet allow to classify all the different, still not well-characterized clinical subtypes of sporadic PD. Moreover, the contribution of environmental influences, chronic exposure to toxins, such as pesticides, to cause PD syndromes in predisposed individuals is still unknown. This specifically concerns advPD patients, as the progression that defines the point, when patients reach this so-called advanced stage of the disease is based on individual differences in disease expression, e.g., with GBA-related PD presenting with more prominent cognitive impairments and axial symptoms related to advPD (Brockmann et al. 2015).

During neurodegeneration in PD, various neuronal death mechanisms occur. Nearly, all of them end up in a cell death cascade of increased oxidative stress, glutamate toxicity the final step of apoptosis. Clinical research on regenerative therapies needs to account for the variety of the PD subtypes and the further probable impact of epigenetic, environmental, toxicological and infectious stressors on onset and progression of PD. Yet, neuroregenerative approaches were only successful in experimental research based on a single pathological process, e.g., by toxin rodent models, e.g., with 6-OH-dopamine, rotenone or 1-methyl-4-phenyl-1,2,3,6-tetrahydropyridine (MPTP) application that rarely model the chronic neuronal cell death particularly in non-dopaminergic systems, and sometimes overestimated the ability of in vitro and in vivo PD models to translate into therapies that delay or prevent advPD stages.

Thus, therapeutic stimulation of endogenous repair mechanisms for affected glial and neuronal cells is urgently needed and not only dopamine substitution in the nigrostriatal system. This approach would well be accepted by patients (even in earlier stages), caregivers and physicians, because it may prevent or reverse the advPD. One candidate for such a more general approach is the modulation of the repulsive guidance molecule A (RGMa). This protein is involved in the physiologic repair mechanisms of neurons. Therapeutic RGMa decrease supports regeneration of lesioned neurons according to experimental findings in various acute and chronic experimental models of nervous system diseases independent of inflammatory, degenerative or ischemic origin (Tao et al. 2013; Demicheva et al. 2015).

Generally, efficacious treatment of advPD implies an intervention-specific risk (e.g., surgical procedure for DBS) and may cause at least temporary adverse effects. Thus, clinicians together with their more and more well-informed patients, respectively caregivers, discuss an individual therapeutic risk benefit ratio. This is the essential precondition to initiate and to perform more personalized therapies.

Currently, clinicians use a certain drug portfolio for amelioration of PD symptoms by establishing an individually balanced and combined drug cocktail. Careful and slow titration with continuous consideration of the tolerability, safety and the needs of the patients and their caregivers is the precondition for a successful treatment of PD in the long term and especially in advPD. However, standardized treatment approaches with guidelines derived from classical clinical trials with highly selected patients may be limited, when heterogeneous subtypes of advPD are treated in clinical practice (Weiner et al. 2009). Clinicians acknowledge that each PD patient is different; however, it requires novel clinical trial designs and further operationalization of stratification criteria to translate this concept into guidelines. This requires more individualized trials as part of the mix and value the observation in single patients.

Already in the past relevant advances in the drug treatment of PD patients were contributed by clinicians and their patients via close observations of clinical symptoms and therapeutic effects. Typical examples are the introduction of levodopa therapy by Birkmayer and Hornykiewicz (1961) or the clinical discovery on the efficacy of amantadine on motor behavior in one PD patient during the treatment of influenza (Schwab et al. 1969). The latter case indicates that observations in single individuals can be still meaningful, e.g., in terms of hypothesis generation, and may be subsequently translated into larger trials. In contrast to former single case reports, the technological advances permit today to perform multiple simultaneous measurements of different biological parameters within one individual at reasonable costs. This is in line with recent initiatives from life sciences funding bodies and governments that increasingly support more targeted treatment approaches and patient empowerment.

**Fig. 1:**
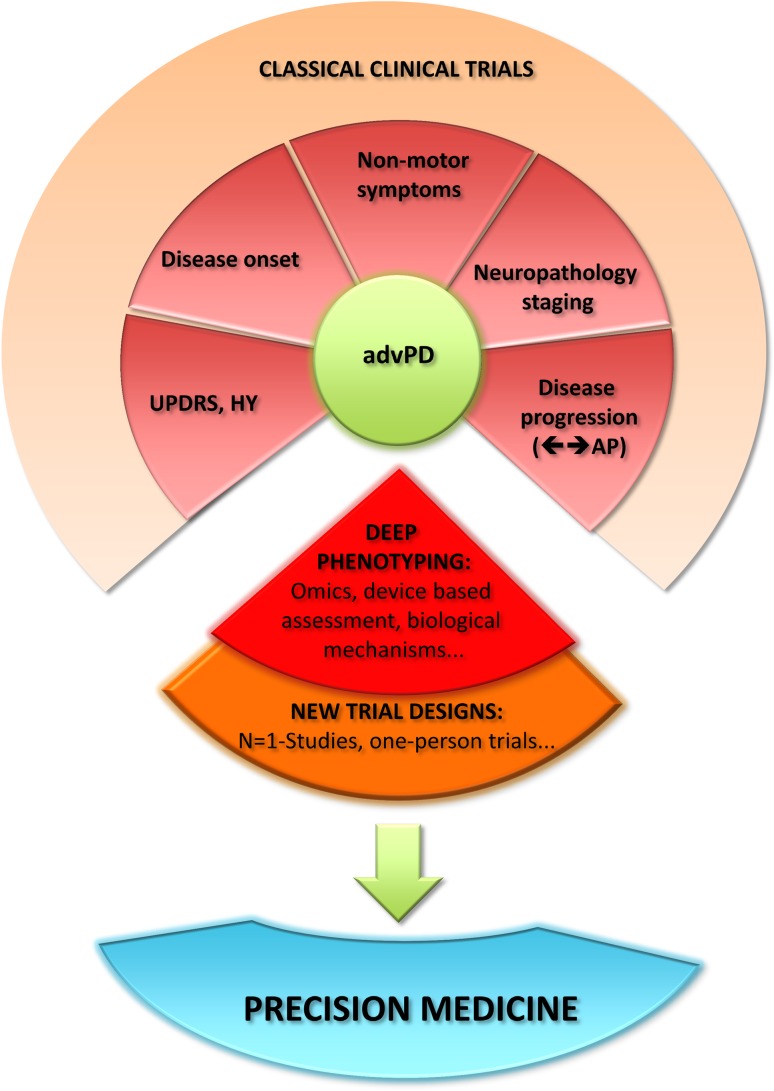
Precision medicine—novel designs for clinical trials. *advPD* advanced Parkinson’s disease, *AP* atypical parkinsonism, *HY* Hoehn&Yahr

Therefore, based on new options for deep phenotyping advPD patients using (1) molecular strategies (‘omics’-based assessment of metabolome, genome, transcriptome, proteome), (2) mobile devices for more objective health data (e.g., accelerometers, smartphone apps) and (3) engagement of patients in medical research, novel designs for clinical trials emerge (Fig. 1). This allows for tailoring dosages to individual metabolic profiles and avoid testing of medication in a large number of unselected patients, typically including a substantial number of non-responders to establish precision medicine (Schork 2015). The underlying, so-called one-person trials focus on individual, not average, therapeutic response and, therefore, account for the whole complexity of different subtypes of advPD. Given such visions, we should be able to implement with much easier protocols, i.e., avoiding drug–drug interactions by introducing individualized drug monitoring especially (but not exclusively) in advPD. These patients are typically not reflected in standard clinical trials and treated with a variety of drugs, e.g., for mental, autonomic and sensory dysfunction, and thus combine multiple drugs with very different pharmacological mechanisms, which may interfere and cause (severe) adverse drug reactions (Hiemke et al. 2011). Thus, future trials will prove the effectiveness of a novel therapy within individual patients and, therefore, treatment benefit will be delineated in the actual participant and account for possible interactions avoiding side effects.
